# Dry Heating of Curcumin in the Presence of Basic Salts Yields Anti-inflammatory Dimerization Products

**DOI:** 10.1021/acsomega.4c03257

**Published:** 2024-08-20

**Authors:** Paula B. Luis, Fumie Nakashima, Sai Han Presley, Gary A. Sulikowski, Claus Schneider

**Affiliations:** † Department of Pharmacology, Vanderbilt University, Nashville, Tennessee 37232, United States; ‡ Department of Chemistry, Vanderbilt University, Nashville, Tennessee 37232, United States; § Vanderbilt Institute of Chemical Biology, Vanderbilt University, Nashville, Tennessee 37232, United States

## Abstract

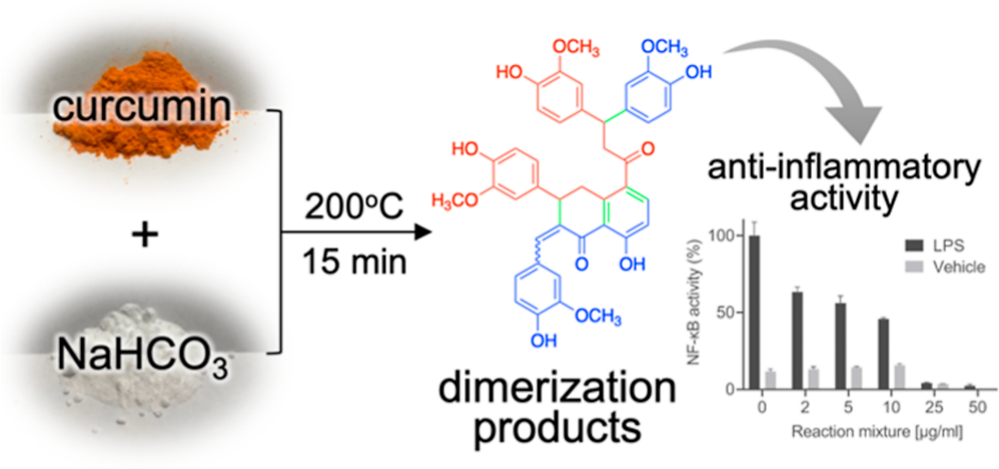

Curcumin exerts some of its biological effects via degradation products formed by spontaneous oxidation at physiological, i.e., weakly basic, pH. Here, we analyzed products formed by dry heating of curcumin in the presence of a basic salt (sodium bicarbonate and others). Under the dry heating conditions employed, curcumin was completely consumed, yielding products entirely different from those obtained by autoxidative degradation in buffer. Bioassay-guided fractionation of the reaction mixture was used to identify and isolate compounds with anti-inflammatory activity in a cell-based assay. This provided two dimers of curcumin, dicurmins A and B, featuring a partly saturated naphthalene core that inhibited lipopolysaccharide-induced activation of NF-κB in RAW264.7 cells. Dicurmin A and B are unusual derivatives of curcumin lacking key functional moieties yet exhibit increased anti-inflammatory activity. The process of dry heating of polyphenols in the presence of a basic salt can serve as a novel approach to generating bioactive compounds.

## 1 Introduction

Extracts from the rhizome of the turmeric plant (Curcuma longa L., Zingiberaceae) are top-selling dietary supplements in the United States.^1^ Turmeric dietary supplements provide curcumin and its analogues demethoxycurcumin (DMC) and bisdemethoxycurcumin (BDMC) as bioactive compounds.^2^ Consumers use turmeric supplements to address health conditions associated with various diseases including pain, inflammation, gastrointestinal complications, and even cancer and neurologic disorders.^3^ The use of turmeric or curcumin in these conditions is paralleled by a large number of clinical trials conducted with curcumin although, despite some notable exceptions, biological or even therapeutic effects have been lacking behind expectations.^4−6^

A major hurdle to wider clinical application of curcumin is considered to be its poor oral bioavailability combined with rapid metabolism.^7^ To overcome pharmacokinetic limitations, a number of formulations have been developed, and many provide increased bioavailability of curcumin in humans.^8,9^ These formulations are increasingly available in the marketplace and are also tested in clinical studies.^5,10^

Besides pharmacokinetic aspects limiting utility of curcumin, there has also been a focus on pharmacodynamics, for example, addressing the role of metabolites of curcumin as mediators of bioactivity.^11,12^ These efforts have shown that some phase-1 and -2 metabolites retain bioactivity, including reduced^13,14^ and conjugated metabolites,^15−17^ with the latter hypothesized to becoming active upon in vivo deconjugation back to curcumin.^18−20^ In addition, oxidative metabolites have been shown to mediate anti-inflammatory and other effects of curcumin.^21−24^ To date, however, studies analyzing oxidative metabolites have been limited to cultured cells, and it is not known whether these products also contribute to the bioactivity of curcumin in vivo.

Curcumin is highly unstable under mildly alkaline conditions, i.e., in aqueous buffer at physiological pH,^25,26^ where it undergoes rapid autoxidation yielding oxidative metabolites.^27^ Autoxidation is initiated by hydrogen abstraction from one of the phenolic hydroxyls in a SPLET process (sequential proton loss followed by electron transfer (SPLET).^28^ A subsequent cascade of radical-mediated reactions yields oxidative metabolites that contribute to the anti-inflammatory activity of curcumin,^22,29^ suggesting that bioactivity might be enhanced by approaches that support the initial hydrogen atom abstraction leading to autoxidation. To that end, we analyzed the effect of basic salts like sodium bicarbonate on the transformation of curcumin and tested whether such treatment results in the formation of novel and bioactive products.

## 2 Materials and Methods

### 2.1 Materials

Curcumin was purchased as a purified turmeric extract labeled “curcumin (mixture of curcumin, DMC, and BDMC)” from Fluka (product number 28260). All other chemicals and solvents were obtained from Sigma-Aldrich or Fisher Chemical at the highest purity available. Medium-chain triglyceride oil from coconut (Better Body Foods, Lindon, UT) was obtained from a local supermarket. The oil contained 13 g of saturated and 0 g each of trans, polyunsaturated, and monounsaturated fat per serving (15 mL).

### 2.2 Dry Heating Reactions

In a standard reaction, commercial curcumin (5 mg) was mixed with sodium bicarbonate (NaHCO_3_; 20 mg) in a glass vial, covered with aluminum foil, and placed in a laboratory oven at 200 °C. After 15 min, the vial was withdrawn from the oven and allowed to cool to room temperature, the content was dissolved by adding acetonitrile (2 mL) and water (1 mL), and an aliquot was analyzed by RP-HPLC. Modifications to the standard reaction mixture used a different type of salt (potassium carbonate, calcium carbonate, ammonium acetate, manganese chloride, manganese-IV-oxide, or copper sulfate; added by weight), different ratios of salt versus commercial curcumin (from 0.125 to 1 to 20 to 1; by weight), addition of a solvent (glycerol or medium-chain triglyceride oil), or different reaction times and temperatures as described in the Results. Reactions in glycerol and medium-chain triglyceride oil used 250 μL of solvent, 5 mg of commercial curcumin, and 20 mg of NaHCO_3_. The reactions in glycerol were heated at 200 °C for 1, 2, 3.5, 5, 10, and 15 min and dissolved in 1 mL each of acetonitrile and H_2_O for HPLC analysis. The reactions in medium-chain triglyceride oil were heated at 200 °C for 15 min and dissolved in 2 mL of acetonitrile and 0.4 mL of H_2_O.

### 2.3 HPLC Analysis

The reaction mixtures were analyzed by using an Agilent 1200 HPLC system equipped with a diode array detector. An aliquot (5 μL) of the dissolved reaction mixtures was injected on a Waters Symmetry C18 5 μm column (4.6 × 250 mm) eluted with a solvent of acetonitrile/water/acetic acid 20/80/0.01 (by volume) changed to 80/20/0.01 (by volume) in 20 min using a linear gradient at a 1 mL/min flow rate. Chromatograms were recorded at 205, 235, and 430 nm.

### 2.4 Fractionation of the Reaction Mixture

A larger-scale reaction of commercial curcumin (100 mg) and NaHCO_3_ (500 mg) was heated at 200 °C for 30 min. Aliquots (100 mg) of the reaction mixture were dissolved in 1 mL of MeOH and 5 mL of H_2_O, and aliquots (100 μL) were injected on semipreparative RP-HPLC using a Thomson Instrument Co. Advantage C18 60 Å 5 μm column (10 × 250 mm) eluted with a linear gradient of acetonitrile/water/acetic acid 20/80/0.01 (by volume) to 80/20/0.0 (by volume) in 25 min at a flow rate of 3 mL/min. Fractions were collected from 2 to 6 min (fraction **A**), 6–11 min (fraction **B**), 11–16 min (fraction **C**), 16–20 min (fraction **D**), and 20–25 min (fraction **E**). Combined fractions from multiple injections were placed in a −20 °C freezer overnight, and the top (acetonitrile) phase was recovered. The solvent was removed using a rotary evaporator, and the residue was dissolved in DMSO for bioassay testing or in MeOH for further HPLC analyses.

Further fractionation of fractions **C**, **D**, and **E** used a Thomson Scientific C18 5 μm column (10 × 250 mm) eluted at a 3 mL/min flow rate and a gradient of acetonitrile/water/acetic acid 40/60/0.01 (by vol.) to 60/40/0.01 (by vol.) in 20 min for fractions **C** and **D** and isocratic elution at 55/45/0.01 (by vol.) for fraction **E**. The peaks or fractions were collected manually, combined from several identical chromatographic runs, and evaporated from the solvent. The fractions were tested at 1:1 and 1:10 dilutions in the bioassay or used for additional purification or product identification.

### 2.5 Bioassay

Stock solutions of the crude reaction mixture as well as isolated compounds were prepared by weighing the dry material, followed by dissolving in an appropriate amount of DMSO. HPLC-collected peaks and fractions were evaporated from the solvent and dissolved in 50 μL of DMSO. Isolated compounds **E5** and **E10** were of >95% purity as determined using RP-HPLC with diode array detection. Stock solutions were stored at −20 °C between experiments. The bioassay used a Ready-to-glow Secreted Luciferase Reporter assay to quantify the effect of the crude reaction product, collected fractions, and purified compounds on lipopolysaccharide (LPS)-induced NF-κB activity. RAW264.7 cells stably transfected with pNFkB-TA-MetLuc with TB vector (Clontech) were maintained with 1 mg/mL geneticin.^23^ Cells were seeded in 24-well plates at 250,000 cells/well in DMEM (500 μL) and incubated overnight. The next day, cells were washed with PBS twice and treated with fractions or compounds at the indicated dilution/concentration in DMEM for 45 min. The amount of DMSO was the same in all experiments. The cells were then stimulated with LPS (100 ng/mL) and after 4 h, an aliquot of the medium (50 μL) was removed for determination of luciferase activity. Bioassay analyses were performed in three independent replicates, and within each replicate, each data point was the mean of three identical wells.

### 2.6 Western Blot Analysis

RAW264.7 cells were cultured in DMEM with 10% FBS and seeded in six-well plates and grown for 24 h. For COX-2 expression, cells were pretreated with compounds for 30 min prior to stimulation with LPS (100 ng/mL) and harvested after 4 h. For inducible nitric oxide synthase (iNOS) expression, cells were pretreated with compounds for 30 min, stimulated with LPS (1 μg/mL), and harvested after 8 h. Cells were washed with PBS and lysed using a lysis buffer (Cell Signaling) containing a protease inhibitor cocktail (Sigma). Cellular protein (5 μg for COX-2; 15 μg for iNOS) was resolved by using 10% SDS-PAGE and transferred to nitrocellulose. Primary antibodies were used for detection of COX-2 (Cayman Chemical no. 160126; 1:2000), iNOS (Cell Signaling no. 13120S; 1:1000), and β-actin (Cell Signaling no. 3700S; 1:3000). Secondary antibodies (926-68021 and 926-32210) from LI-COR Biosciences were used at a 1:20000 dilution. Western blots were repeated three times, independently, with similar results.

### 2.7 Quantification of Nitric Oxide (NO)

RAW 264.7 cells were seeded in a 24-well plate at 400,000 cells/well in complete media and incubated at 37 °C overnight. The cells were pretreated with **E5** and **E10** for 30 min and then stimulated with LPS at a final concentration of 1 μg/mL for 8 h. Nitrite was quantified in the cell culture medium as an indicator of nitric oxide (NO) levels. The cell culture medium supernatant (80 μL) was incubated with Griess reagent (1% sulfanilamide and 0.1% naphthylethylenediamine dihydrochloride in 2.3% H_3_PO_4_; 50 μL) for 10 min. Absorbances were read at 560 nm, and the nitrite concentration was calculated by using a sodium nitrite calibration curve.

### 2.8 Quantification of IL-1β and IL-6

RAW 264.7 cells were seeded in a 24-well plate at 400,000 cells/well in complete media and incubated at 37 °C overnight. The cells were pretreated with **E5** and **E10** for 30 min and then stimulated with LPS (1 μg/mL) for 4 h. IL-1β and IL-6 were quantified in the cell supernatants using Quantikine ELISA kits from R&D Systems.

### 2.9 High-Resolution Mass Spectroscopy

Samples were directly infused (10 μM at 10 μL/min) into a Thermo Scientific LTQ XL Orbitrap instrument operating in electrospray ionization (ESI) negative mode. Prior to the analysis, the instrument was calibrated with an ESI-negative ion calibration solution.

### 2.10 Nuclear Magnetic Resonance

NMR spectra were recorded by using a Bruker AV-II 600 MHz spectrometer equipped with a cryoprobe. Chemical shifts (δ value) are given relative to the residual nondeuterated solvent and are reported in parts per million (ppm). Coupling constants (*J*) are given in hertz (Hz). Pulse frequencies were taken from the Bruker library.

## 3 Results

### 3.1 Source of Curcumin

The source of curcumin was a purified turmeric extract, which is the most widely used form of curcumin. The extract consisted of curcumin (75%), DMC (21%), and BDMC (4%), with relative abundances established by RP-HPLC analysis. The product is termed “commercial curcumin” here.

### 3.2 Dry Heating of Commercial Curcumin

**Figure 1:**
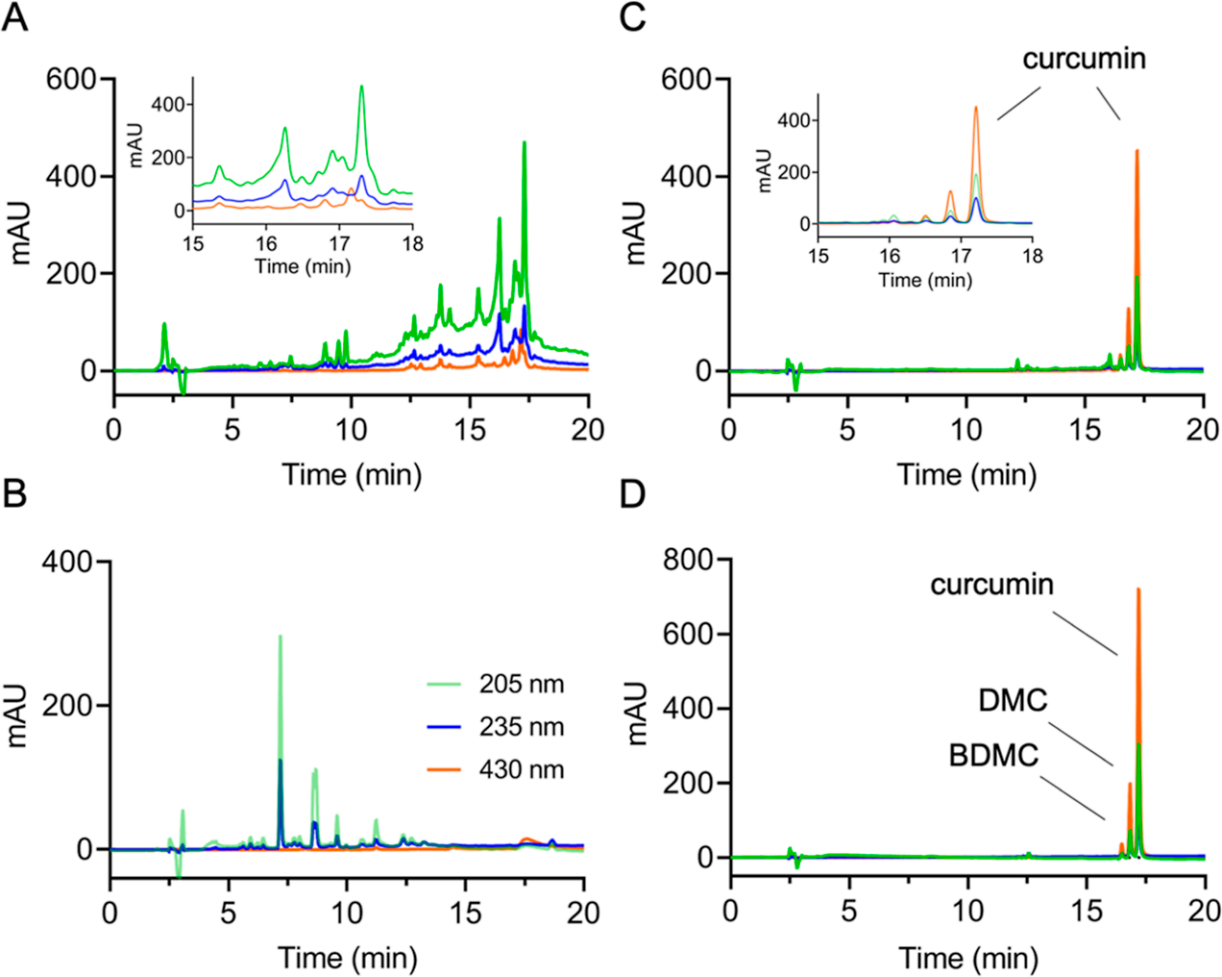
Dry heating of commercial curcumin in the absence and presence of NaHCO_3_. RP-HPLC analysis of a (A) mixture of commercial curcumin and NaHCO_3_ (1:3, w/w) heated at 200 °C for 15 min, (B) autoxidation of pure curcumin at pH 7.5 (60 min; room temperature), (C) commercial curcumin heated at 200 °C for 15 min, and (D) commercial curcumin starting material. The insets in panels A and C show an expanded view of the chromatograms between 15 and 18 min retention time. Chromatograms shown were recorded at 205 nm (green), 235 nm (blue), and 430 nm (orange) using a diode array detector.

The effect of the basic salt in inducing transformation of curcumin was apparent in RP-HPLC analyses comparing the commercial curcumin starting material with reaction products of autoxidative degradation of pure curcumin in pH 7.5 buffer and products obtained after heating the starting material as a dry powder in the absence or presence of NaHCO_3_ at 200 °C for 15 min. The presence of NaHCO_3_ resulted in quantitative transformation of the curcuminoids (Figure 1A) as was apparent from the decreased absorbance at 430 nm of the peaks eluting at a retention time of around 17 min. Transformation in the presence of NaHCO_3_ gave a product profile markedly different from that of autoxidation conducted in buffer at physiological pH (Figure 1B). In contrast, heating in the absence of salt did not result in abundant product peaks, even though the starting material was decreased by about 40% (Figure 1C,D). This was compatible with formation of volatile and possibly other products that were not recovered in the heating reaction.^30^ The large number of reaction products formed in the presence of the basic salt (Figure 1A) made indiscriminate isolation and identification of all products an overwhelming task and was not attempted. Instead, focus was placed on products with potential biological activity.

**Figure 2:**
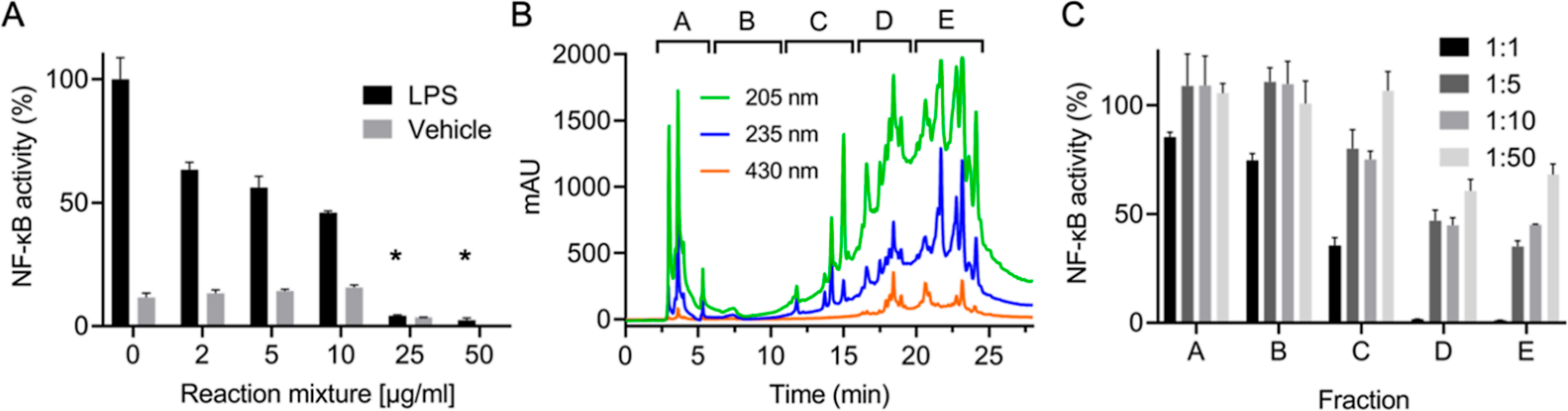
Bioactivity and initial fractionation of the crude reaction mixture. (A) Effect of the reaction mixture (commercial curcumin/NaHCO_3_ 2:1 w/w; 30 min at 200 °C) on NF-κB activity in RAW264.7 cells with (black bars) and without (gray bars) LPS stimulation. (B) A heated commercial curcumin/NaHCO_3_ (1:5, w/w) reaction mixture was injected on a semipreparative RP-HPLC column and 5 fractions were collected (fraction **A**: 2–6 min, **B**: 6–11 min, **C**: 11–16 min, **D**: 16–20 min, and **E**: 20–25 min). (C) The fractions were tested at four dilutions for inhibition of LPS-induced NF-κB activation in RAW264.7 cells. *p < 0.01 versus vehicle in one-way ANOVA.

In order to test for an overall anti- or proinflammatory effect, the crude product mixture was dissolved in DMSO and tested at different concentrations in the bioassay. The reaction mixture was prepared by heating a 2:1 (w/w) mixture of commercial curcumin and NaHCO_3_ at 200 °C for 30 min. The decreased amount of salt did not change the reaction products (see below) and was chosen in order to enhance the solubility of the reaction mixture in DMSO for testing. The bioassay employed RAW264.7 cells stably transfected with an NF-κB response element driving expression of luciferase.^23^ Curcumin and curcuminoids inhibit expression of NF-κB-induced luciferase upon stimulation of cells with LPS^22^ which was taken as an anti-inflammatory effect. RAW264.7 cells were also treated with the crude reaction product in the absence of LPS stimulation in order to test for a proinflammatory effect. The crude reaction product dose-dependently inhibited LPS-induced NF-κB activation in RAW264.7 cells and did not activate NF-κB in cells not stimulated with LPS, suggesting an anti-inflammatory effect of the product mixture (Figure 2A). LPS-induced NF-κB activity was decreased by about half using between 10 and 25 μg/mL of the crude reaction product. For comparison, the IC_50_ value for the commercial curcumin starting material in the same assay was 15 μM which is equivalent to 6 μg/mL.^22^ The crude reaction product was also tested in an MTT assay, showing that viability of RAW264.7 cells was not decreased at concentrations up to 50 μg/mL and treatment for 4 h (data not shown). Thus, the crude mixture of the reaction products was almost as potent as the starting material in inhibiting the NF-κB activity.

### 3.3 Bioassay-Guided Fractionation

In order to isolate and identify products with anti-inflammatory activity, we tested fractions obtained from RP-HPLC separation of the crude reaction product in the cellular NF-κB assay. Semipreparative RP-HPLC provided 5 initial fractions **A**–**E** as shown in Figure 2B. Testing of crude fractions **A**–**E** at 4 dilutions in the cell-based assay indicated the presence of active compounds in fractions **C**, **D**, and **E** (Figure 2C).

**Figure 3:**
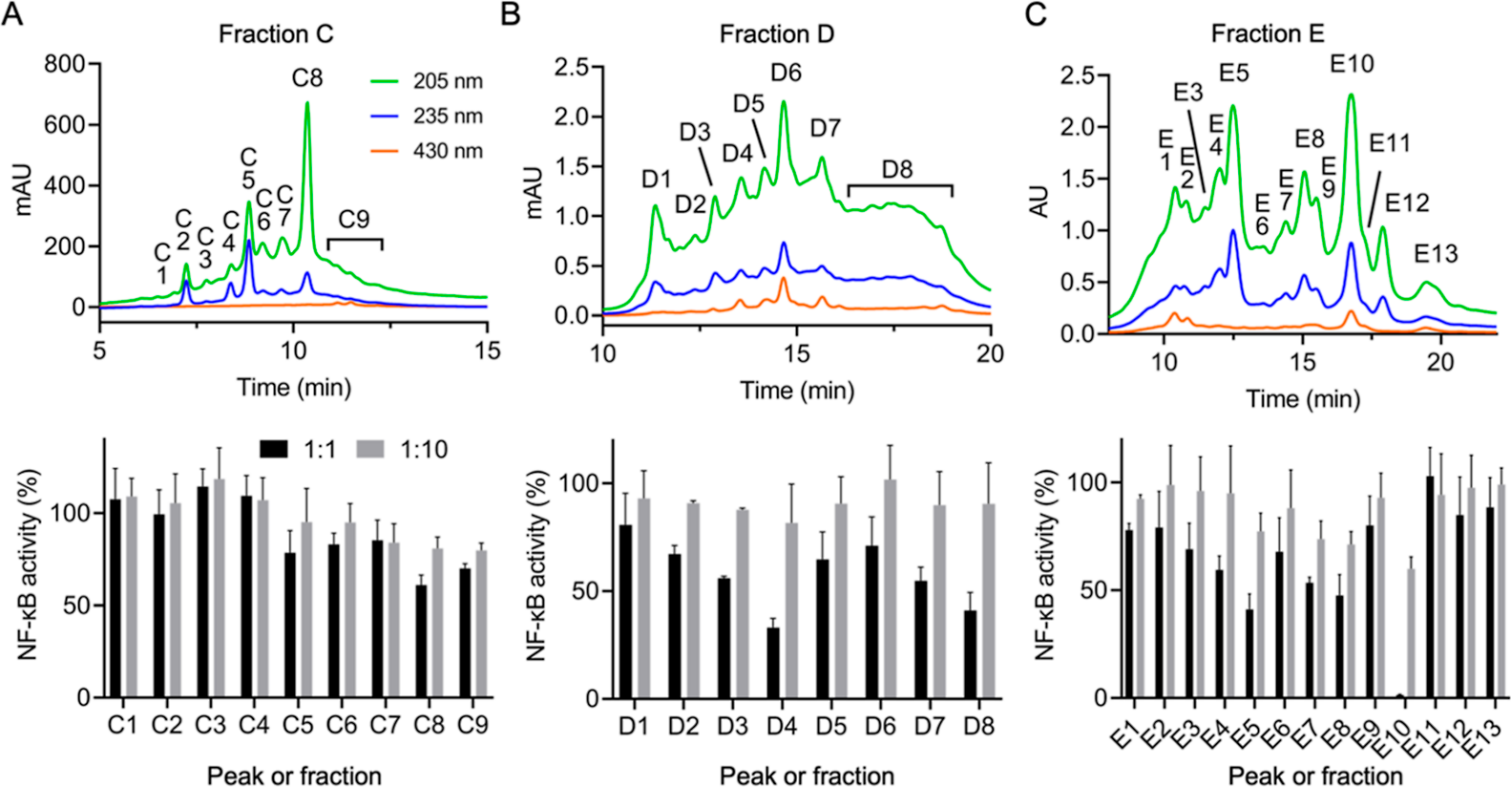
Secondary fractionation and bioactivity of fractions **C**, **D**, and **E**. Semipreparative RP-HPLC separation (top) and bioactivity (bottom) of collected peaks for (A) fraction **C**, (B) fraction **D**, and (C) fraction **E**. Fractions were tested at 1:1 (black bars) and 1:10 dilution (gray bars) for inhibition of LPS-induced NF-κB activation in RAW264.7 cells.

Fractions **C**, **D**, and **E** were further resolved into peaks or fractions, as appropriate, and tested in the cellular assay. Each peak or fraction was tested at 1:1 and 1:10 dilution regardless of peak size since, due to low amount of material obtained, normalization by weight was not feasible. The use of two dilutions was chosen in order to increase confidence that a true effect was observed. Peaks **C1** through **C9** derived from fraction **C** did not show a strong inhibitory effect on NF-κB and therefore were not further analyzed since the inhibitory activity observed in the crude fraction could not be associated with a defined peak (Figure 3A). Fraction **D** was resolved into 8 peaks, with the strongest inhibitory activity in peak **D4** and fraction **D8** (Figure 3B). Fraction **E** gave 13 peaks, with notable activity in peaks **E4**, **E5**, **E7**, **E8**, and **E10** (Figure 3C).

### 3.4 Identification of Active Compounds

**Figure 4:**
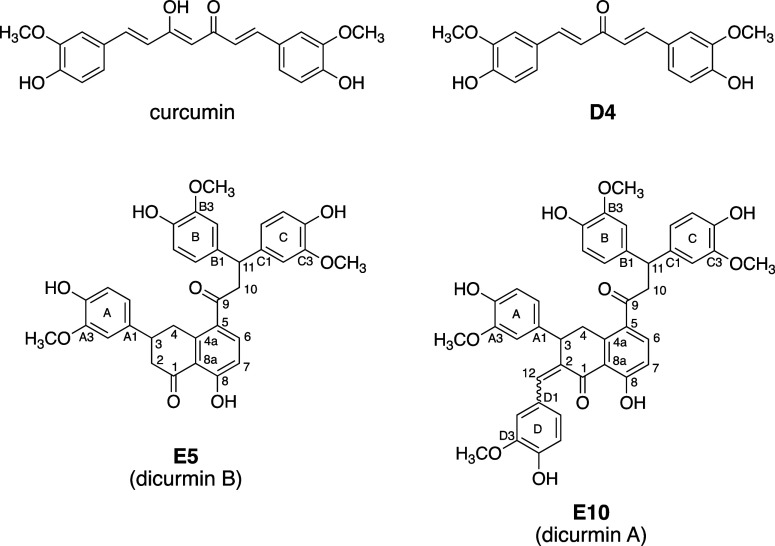
Chemical structures of curcumin and compounds **D4** (deketene curcumin), **E5** (dicurmin B), and **E10** (dicurmin A) formed upon dry heating of curcumin in the presence of NaHCO_3_.

Active peak **D4** was further purified, and its structure was determined using LC–MS and NMR analyses. **D4** was identified as a monocarbonyl analogue of curcumin [1,5-bis(4-hydroxy-3-methoxyphenyl)-1,4-pentadien-3-one; “deketene curcumin”] that previously had been prepared by chemical synthesis^31^ and that was also isolated from pyrolysis reactions of curcumin^32^ and as a natural product from *Curcuma domestica*(33) (Figure 4). **D8** was a fraction of about 3 min eluting at the end of the chromatogram and was not further investigated since it did not contain a distinguishable chromatographic peak upon reanalysis using RP-HPLC.

Among the active peaks in fraction **E**, we focused on peaks **E5** and **E10** since these were the most abundant and amenable to further purification and identification. RP-HPLC separation of peaks **E4**, **E7**, and **E8** showed that each peak consisted of multiple products and activity was not clearly associated with a defined peak (data not shown). Due to the low amount of material for many of the peaks, it was not possible to normalize activity in the bioassay by weight and this may have resulted in missing compounds high in bioactivity but low in abundance.

LC–HR-MS analyses recorded in negative ion mode gave an exact mass of 583.1976 for **E5** consistent with a molecular formula of C_34_H_32_O_9_ (calculated exact mass = 583.1974; Δ = 0.3 ppm). **E10** had an exact mass of 717.2344 providing a formula of C_42_H_38_O_11_ (calculated exact mass = 717.2341; Δ = 0.4 ppm). The molecular formula suggested that **E10** was a dimer of curcumin formed under the loss of water. **E5** also appeared to be a dimer that had lost a moiety comprised of C_8_H_6_O_2_ in addition to water.

**Figure 5:**
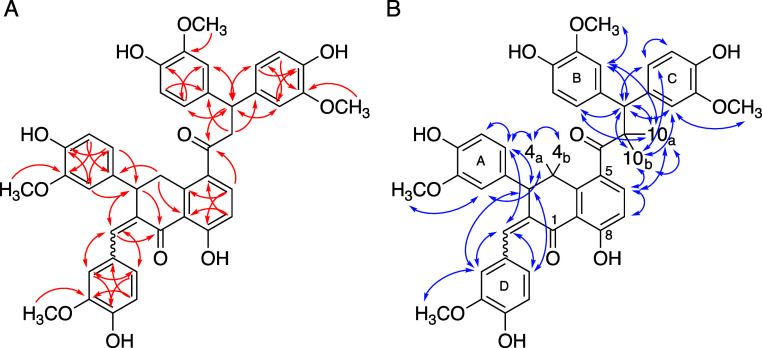
(A) Three-bond HMBC (from ^1^H to ^13^C) and (B) NOESY correlations for **E10** (dicurmin A).

NMR analyses using ^1^H, H, H–COSY, HSQC, HMBC, NOESY, and ^13^C NMR experiments established the structures of **E5** and **E10** (Supporting Information Tables S1 and S2; Figures 4 and 5). Both compounds were identified as dimers of curcumin, with dimerization occurring under the loss of water. **E5**, in addition, also had lost a 4-hydroxy-3-methoxybenzylidene ring (C_8_H_6_O_2_). Analysis of the 3-bond couplings in the HMBC spectra enabled us to identify the central bicyclic core of both compounds as a partially saturated naphthalene derived from apparent dimerization of the heptadienedione moieties of two curcumin monomers (Figure 5A). Dimerization was accompanied by migration of one of the phenolic rings in both **E10** and **E5**. The configuration of the exocyclic double bond in **E10** was not unequivocally established but is likely Z, based on a NOESY signal between H12 and H6 of the A ring (Figure 5B). However, the data do not exclude the possibility that dicurmin A is a mixture of two double bond isomers that were not resolved by HPLC and had identical NMR data. Compounds **E10** and **E5** represented novel structures and were named “dicurmin A” for **E10** and “dicurmin B” for **E5**, respectively.

Structural identification of **E5** and **E10** enabled determination of the molarity of the compounds in a dose-response analysis for inhibition of NF-κB activity in the RAW264.7 cell model. **E5** and **E10** inhibited NF-κB with IC_50_ values of 11.5 μM and 3.4 μM, respectively, while curcumin had an IC_50_ of 18 μM using the same model.^22^

Since the identified products **D4**, **E5**, and **E10** were derived from curcumin, we wanted to test whether similar products were also formed from DMC or BDMC, since the latter curcuminoids were also present in the commercial curcumin starting material. We predicted molecular ions for pure or mixed dimers consisting of various combinations of curcumin, DMC, and BDMC that would be analogous to those of **E5** and **E10**. LC–MS analyses did not show formation of the respective dimers containing DMC or BDMC. Whether the apparent lack of dimerization was due to the lower abundance of DMC and BDMC in commercial curcumin, due to differences in their chemical reactivity or due to formation of dimers not considered in our predictions was not analyzed.

### 3.5 Variation of Reaction Conditions

**Figure 6:**
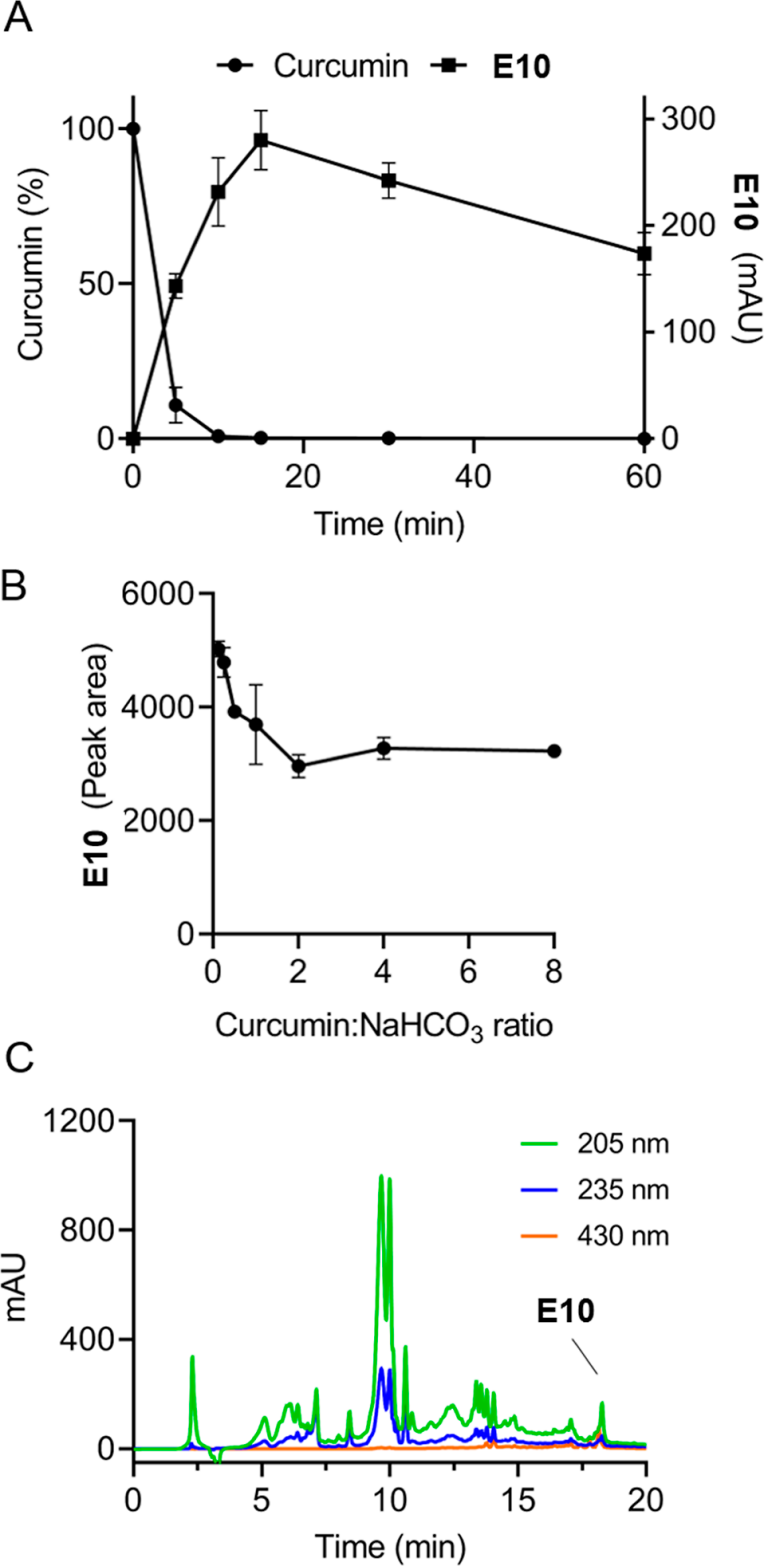
Variation of reaction conditions. (A) Time dependence of the formation of **E10** (commercial curcumin/NaHCO_3_ 1:5 w/w, 200 °C), expressed as peak height (mAU) at 205 nm in RP-HPLC analyses. The amount of curcumin remaining after the reaction is shown in % relative to the starting amount. (B) Dependence of the formation of **E10** on the amount of salt used in the heating reactions (200 °C, 15 min). The ratio of commercial curcumin/NaHCO_3_ (w/w) was varied from 0.125:1 to 1:8. Mean peak areas for **E10** at 205 nm determined in RP-HPLC analyses of two independent reactions are shown. (C) RP-HPLC analysis of a heating reaction (commercial curcumin/NaHCO_3_ 1:5 w/w, 200 °C for 3.5 min) conducted in glycerol.

We tested how modifying the reaction conditions affected the consumption of curcumin and the yield of the major bioactive product **E10** (dicurmin A). A reaction conducted at 150 °C for 15 or 30 min did not result in the formation of the identified bioactive products. Increasing the temperature to 180 °C and using the same reaction times gave chromatograms similar to those at 200 °C. Use of other basic salts like K_2_CO_3_ and NaCO_2_CH_3_ resulted in product profiles similar to NaHCO_3_, whereas CaCO_3_ did not. Salts like MnCl_2_ and MnO_2_ did not induce significant transformation of curcumin when used under similar conditions (1:1, w/w; 200 °C, 15 min), while CuSO_4_ resulted in near quantitative consumption of curcumin though not yielding the dimers **E5** or **E10** (data not shown). The influence of reaction time on the formation of **E10** at 200 °C in the presence of NaHCO_3_ (1:4 w/w) is quantified in Figure 6A indicating that reaction times between 10 and 30 min gave the highest yields.

The amount of salt was varied from 0.125 to 8 times (w/w) relative to commercial curcumin (Figure 6B). The lowest relative amount of salt gave the highest yield of **E10**, which was increased by about 60% compared to a ratio of 1:1 to 1:8 commercial curcumin/NaHCO_3_ (w/w). The highest amounts of salt tested (1:20; not shown) markedly inhibited the transformation of curcumin and formation of **E10**. The highest yield of **E10** (dicurmin A) was about 4% relative to the starting amount of curcumin in commercial curcumin. **E10** was the most abundant peak in the chromatogram shown in Figure 1A, eluting at a 17.3 min retention time, and it was the most abundant product of the transformation reaction.

When commercial curcumin and NaHCO_3_ (1:3 w/w) were dissolved in glycerol prior to heating at 200 °C, complete degradation of curcumin had occurred after 3.5 min reaction time (Figure 6C). Even though the product profile was markedly different from the dry heating reactions, it showed formation of **E10** albeit in low amount. When medium-chain triglyceride oil was used as the solvent and the reaction was heated at 200 °C for 15 min, curcumin was recovered largely unchanged. Only a few products were observed and these appeared different from those formed upon dry heating. No further analysis of the products formed in the presence of a solvent was performed.

### 3.6 Inhibition of iNOS and COX-2 Expression in RAW264.7 Cells

**Figure 7:**
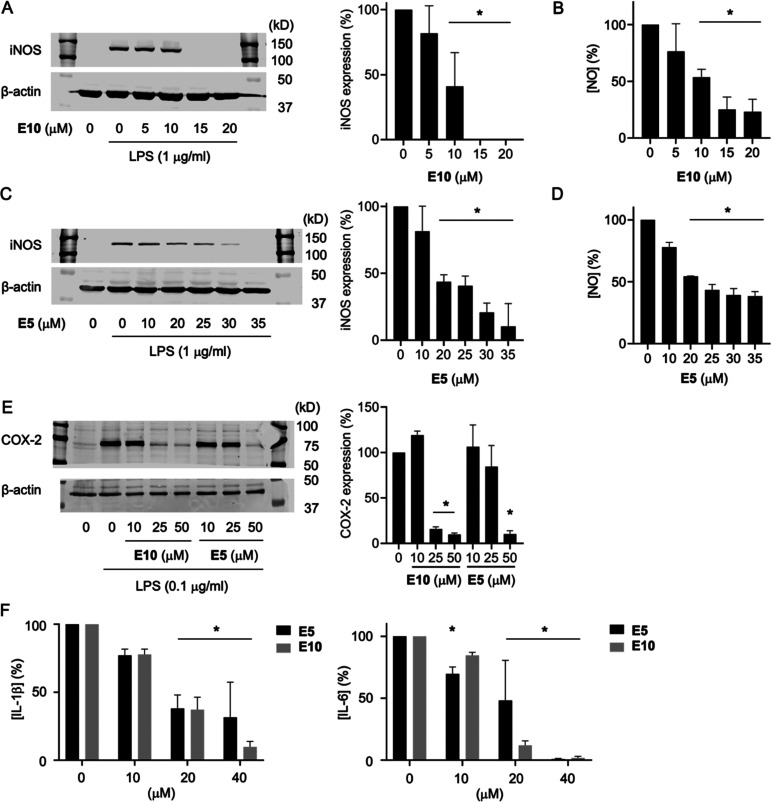
Anti-inflammatory effects of **E10** (dicurmin A) and **E5** (dicurmin B) in activated RAW264.7 cells. (A) Western blot detection and quantification of expression of iNOS and (B) NO in LPS-activated RAW264.7 cells treated with **E10**. (C) Western blot detection and quantification of iNOS and (D) NO in LPS-activated RAW264.7 cells treated with **E5**. (E) Western blot detection and quantification of LPS-induced expression of cyclooxygenase-2 (COX-2) in RAW264.7 cells treated with **E10** and **E5**. (F) Quantification of cytokines IL-1β and IL-6 in the supernatants of RAW264.7 cells treated with LPS and **E10** (black bars) and **E5** (gray bars). *p < 0.01 versus vehicle in one-way ANOVA.

In order to corroborate the results from the luciferase activity assay, we tested whether the crude reaction mixture and isolated peaks were able to inhibit expression of target proteins of NF-κB induced by treatment of RAW264.7 cells with LPS. The cells were pretreated with the purified compounds **E5** and **E10** and activated with LPS. **E5** and **E10** dose-dependently decreased the LPS-induced expression of iNOS and its NO product (Figure 7A–D) as well as the enzyme cyclooxygenase-2 (Figure 7E). Both compounds also decreased the release of LPS-induced cytokines IL-1β and IL-6 in the RAW264.7 cell supernatants (Figure 7F).

## 4 Discussion

The degradation of curcumin in aqueous solution at physiological pH proceeds as an autoxidation^27^ and yields a series of products most of which contain a characteristic cyclopentadione carbocycle core.^29^ The final bicyclopentadione product of curcumin autoxidation appears biologically inert,^34^ in contrast to unstable reaction intermediates that engage in electrophilic protein binding with redox-sensitive protein cysteines as a means to achieving a biological effect.^21,24,29,35,36^ Of interest here is the fact that autoxidation–degradation requires an aqueous medium at basic pH. Under these conditions, phenol deprotonation is facile and followed by electron transfer from the phenolate to O_2_ in a SPLET mechanism leading to a phenoxy radical intermediate.^28,29^ The latter undergoes rapid cyclization, leading to a cyclopentadione radical intermediate. Addition of O_2_ followed by hydrogen atom transfer from a second curcumin molecule continues the autoxidation and affords the final bicyclopentadione product.^29^

Interestingly, none of the autoxidation products were observed in the dry heating reaction of commercial curcumin with basic salts, even though transformation was clearly dependent on the basic character of the salts used (Figure 1). Instead, two of the identified products, dicurmins A and B, were formed by dimerization of two curcumin molecules, and the initial dimerization step likely proceeded via base-catalyzed aldol condensation.

**Figure 8:**
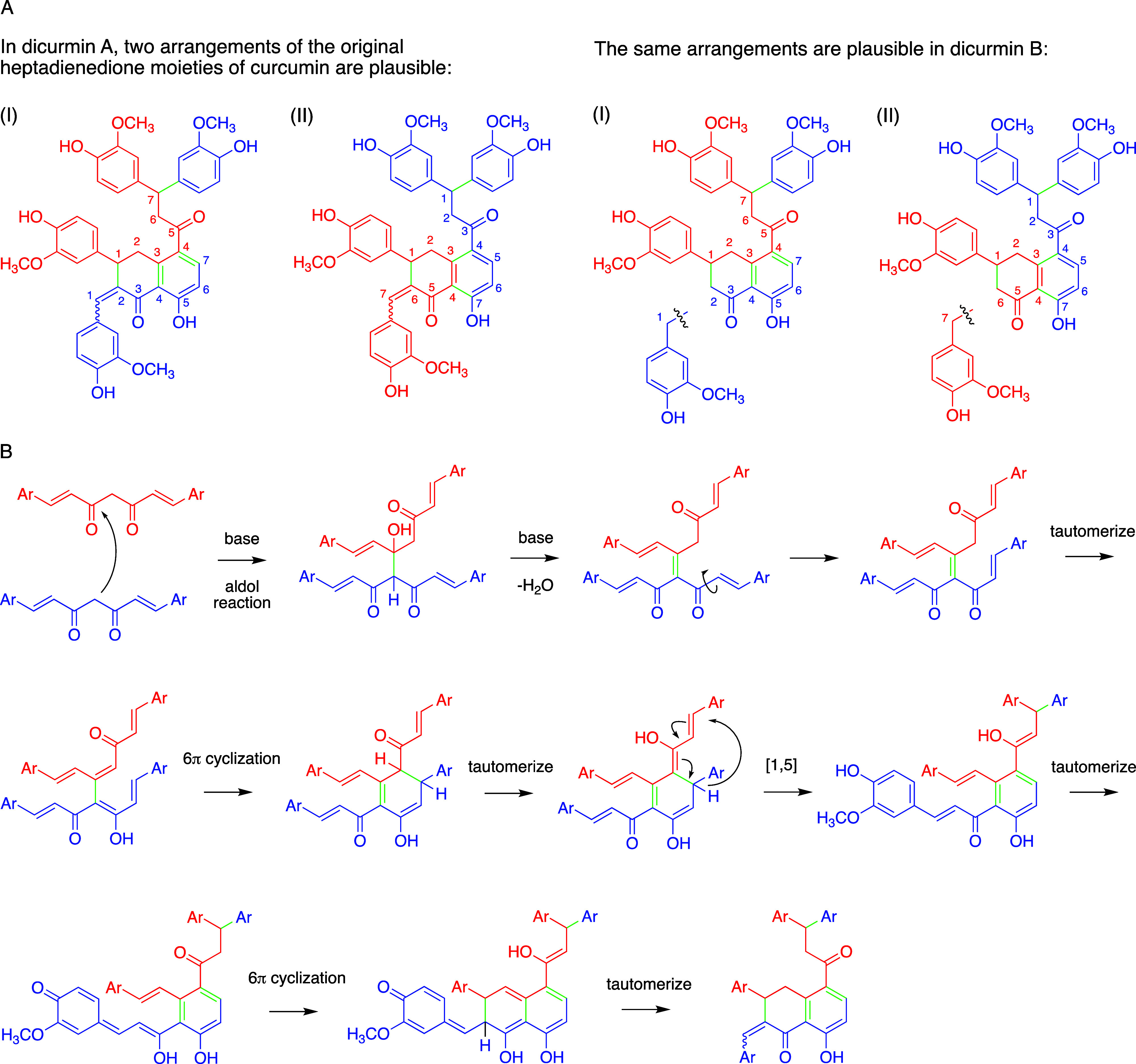
Proposed mechanism of formation of dicurmin A and B. (A) Arrangements (I) and (II) of curcumin monomers appear possible in the formation of the dimeric products. (B) Proposed aldol condensation mechanism of formation of dicurmin A compatible with heptadienedione arrangement (I). For simplicity, only the heptadienedione moieties are drawn and methoxyphenol rings are abbreviated (Ar). The original curcumin monomers are shown in red and blue, respectively, and newly formed carbon–carbon bonds are in green. The atom numbering refers to the carbons in the heptadienedione moiety of curcumin and differs from the numbering used in Figure 4.

Structural examination of dicurmins A and B reveals two possible orientations of curcumin monomers, leading to dimerization (Figure 8A). While direct evidence for either arrangement during dimerization is lacking, arrangement (I) is compatible with the reaction pathway shown in Figure 8B. According to this proposal, aldol condensation sets the stage for a series of pericyclic transformations terminating in dicurmin A. Aldol condensation was followed by bond rotation and tautomerization to facilitate 6π-electrocyclization. A [1,5]-aryl migration of a guaiacol moiety led to aromatization of the newly formed ring. A second series of tautomerization and 6π-electrocyclization gave dicurmin A. Formation of dicurmin B most likely follows the same reaction sequence but includes loss of a 4-hydroxy-3-methoxybenzylidene moiety, possibly involving further oxidation of the quinone methide when undergoing the second 6π-cyclization. Formal hydration of dicurmin A benzylidene followed by a retro aldol fragment would afford dicurmin B and 4-hydroxy-3-methoxybenzylaldehyde. The oxidation state of the fragment or its identity has not been established.

From a structure–activity perspective, there are fundamental differences between the dicurmins and curcumin. The β-dicarbonyl of curcumin enables metal binding and presents an acidic 1,3-diketone hydrogen. The phenolic hydroxyl with extended conjugation into the heptadienedione moiety is responsible for the antioxidant effects, as well as its autoxidation to form reactive electrophiles. The dicarbonyl element is missing in dicurmin A and B. Both compounds appeared chemically stable compared to curcumin; i.e., they are unlikely to yield protein binding electrophiles. It was thus surprising that the novel dimers exerted anti-inflammatory effects via inhibition of NF-κB with a slightly higher potency than curcumin. Curcumin targets redox-active cysteine residues in IKKβ and NF-κB as an inhibitory mechanism^23,37^ but it is unlikely that the dicurmins engage in similar protein binding since they appear much less electrophilic. There are other mechanisms by which the NF-κB pathway is targeted by natural products, for example, via inhibiting dimerization of the upstream Toll-like receptors but this inhibition is also mediated by electrophilic protein binding.^38^ The target(s) of dicurmins A and B in mediating the inhibition of NF-κB remains to be elucidated.

The generation of bioactive compounds by thermal transformation of curcumin has been of interest in the past. Dahmke and co-workers describe 20 min pyrolysis reactions of commercial curcumin conducted at 250 °C in the absence or presence of olive oil or coconut fat.^32^ In the presence of the lipid component, deketene curcumin (peak **D4** in our analyses) was an abundant product, and it was the only product the authors identified by isolation and structural analysis using NMR.^32^ The biological and antiproliferative activities of deketene curcumin have been well-studied,^31,39^ and it has been speculated that some of the biological effects of dietary turmeric when provided through traditionally prepared dishes are mediated by this compound.^32^ Other products described by Dahmke and co-workers were only tentatively assigned based on their *m*/*z* values in LC–MS analyses, including isomers of curcumin, DMC, and BDMC, as well as two dimeric products with MW 718 and 734.^32^ One of the putative dimers described by Dahmke and co-workers shares the same MW (718) with dicurmin A but it is impossible to determine whether the two products might be identical due to the very limited information available in ref (32). In a different study, heating reactions were conducted at 180 °C for 5–70 min and showed rapid consumption of curcumin accompanied by formation of cleavage products like ferulic acid, 4-vinyl guaiacol, vanillin, and vanillic acid, while no reference was made to higher molecular weight products.^30^ Longer heating reactions (2 h at 120–210 °C) in the absence of an additional reagent yielded compounds described as polymeric “curcumin-derived carbon quantum dots” with potent antiviral effects.^40^ The most potent antiviral products were obtained when curcumin was heated at 180 °C but there is little information regarding the chemical structure of the active compound(s) formed.^40^ In conclusion, the high-temperature treatment of curcumin, in the absence or presence of additional reagents, gives products with interesting biological activities. Heating of curcumin in the presence of basic salts adds to the diversity of bioactive derivatives by yielding the anti-inflammatory dimerization products dicurmins A and B.

## Supporting Information Available

Tabulated NMR spectra of compounds **E5** and **E10** (PDF)

## Abbreviations

BDMC: bisdemethoxycurcumin
COX-2: cyclooxygenase-2
DMC: demethoxycurcumin
IKKβ: inhibitor of κB kinase β
iNOS: inducible nitric oxide synthase
LPS: lipopolysaccharide
NF-κB: nuclear factor κB
SPLET: sequential proton loss electron transfer.
