# Supplementary material for: Dry Heating of Curcumin in the Presence of Basic Salts Yields Anti-inflammatory Dimerization Products
Source: ACS Omega. 2024 Aug 20;9(35):37025–34. doi: 10.1021/acsomega.4c03257 (PMC11375705; doi:10.1021/acsomega.4c03257)
Supplement: Supplementary file 1 — ao4c03257_si_001.pdf [file ao4c03257_si_001.pdf]

Supporting Information

Dry heating of curcumin in the presence of basic salts yields anti-inflammatory dimerization products

Paula B. Luis[1], Fumie Nakashima[1,#], Sai Han Presley[1], Gary A. Sulikowski[2,3], and Claus Schneider[1,3*]

[1]Department of Pharmacology, [2]Department of Chemistry, and [3]Vanderbilt Institute of Chemical Biology, Vanderbilt University, Nashville, TN 37232, U.S.A.

Supplementary Table 1.
NMR spectroscopic data (600 MHz, $CDCl_3$) for **E5** (dicurmin B; 5-(11,11-bis(4-hydroxy-3-methoxyphenyl)propanoyl)-8-hydroxy-3-(4-hydroxy-3-methoxyphenyl)-3,4-dihydronaphthalen-1(2*H*)-one).

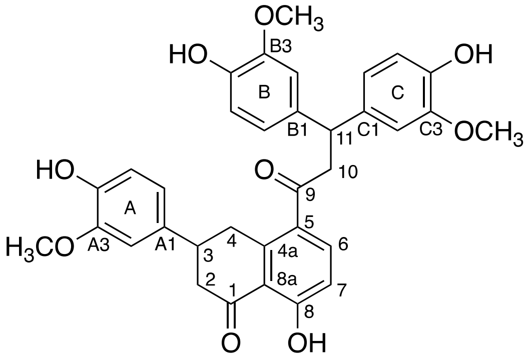

position	δ_C, type	δ_H (J in Hz)	COSY	HMBC	NOESY
1	205.4, C	-			
2_a	45.7, CH_2	2.87, dd (2.2; 16.1)	3	1, 3, 4a, 8a	3, A2, A6
2_b	45.7, CH_2	2.80, dd (13.2; 16.0)			3, A2, A6
3	40.0, CH	2.97, m	2, 4_a, 4_b	1	2_a, 2_b, 4, A2, A6
4	35.7, CH_2	2.91, m	3, 4_a, 4_b	1, 3	3, A2, A6
4a	146.1, C	-			
5	129.7, C	-			
6	135.9, CH	7.74, d (8.6)	7	4a, 5, 8, 8a, 9	7, 10_a, 10_b, 11
7	115.5, CH	6.89, d (9.8)	6	1, 8, 8a	6
8	165.3, C	-			
8a	118.0, C	-			
9	201.3, C	-			
10_a	48.2, CH_2	3.56, dd (7.8; 15.5)	10_b, 11	9, 11, B1, C1	10_b, 11, B2, C2, B6, C6
10_b	48.2, CH_2	3.42, dd (8.0; 15.8)	10_a, 11	9, 11, B1, C1	10_a, 11, B2, C2, B6, C6
11	46.7, CH	4.48, t (7.8)	10_a, 10_b	9, 10, B1, B2, B6, C1, C2, C6	6, 10_a, 10_b, B2, C2, B6, C6
A1	134.7, C	-			
A2	109.3, CH	6.66, br s		3, A6	2_a, 2_b, 3, 4, OCH_3 (A3)
A3	146.4, C	-			
A4	145.1, C	-			

A5	114.5, CH	6.87, d (10.0)	A6	A1, A3, A4	A6
A6	119.3, CH	6.66, br s	A5	3, A2	2_a, 2_b, 3, 4, A5
B1	135.4, C	-			
B2	110.6, CH	6.62, br s		11, B6	OCH_3 (B3)
B3	146.5, C	-			
B4	144.6, C	-			
B5	114.3, CH	6.78, d (8.3)	B6	B1, B3, B4	B6
B6	119.8, CH	6.66, br s	B5	11, B4	B5
C1	135.7, C	-			
C2	110.7, CH	6.62, br s		11, C6	OCH_3 (C3)
C3	146.5, C	-			
C4	144.2, C	-			
C5	114.1, CH	6.76, d (8.2)	C6	C1, C3, C4	C6
C6	112.0, CH	6.66, br s	C5	11, C4	C5
OCH_3 (A3)	56.0, CH_3	3.89, s		A3	A2
OCH_3 (B3)	55.9, CH_3	3.76, s		B3	B2
OCH_3 (C3)	55.8, CH_3	3.71, s		C3	C2

Supplementary Table 2.
NMR spectroscopic data (600 MHz, CD_3OD) for **E10** (dicurmin A; 5-(11,11-bis(4-hydroxy-3-methoxyphenyl)propanoyl)-8-hydroxy-2-(4-hydroxy-3-methoxybenzylidene)-3-(4-hydroxy-3-methoxyphenyl)-3,4-dihydronaphthalen-1(2*H*)-one).

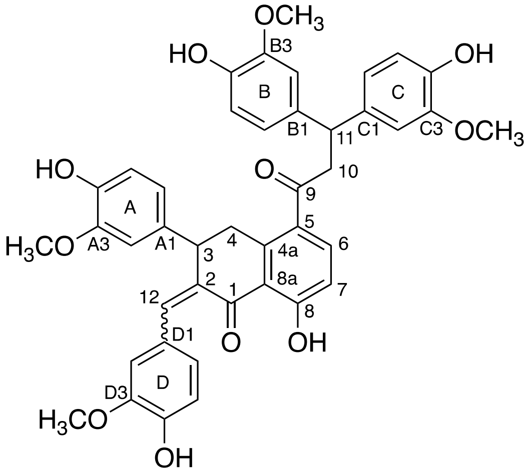

position	δ_C, type	δ_H (*J* in Hz)	COSY	HMBC	NOESY
1	194.6, C	-			
2	135.1, C	-			
3	42.9, CH	4.63, br s	4_b	1, 2, 4, 12	A2, A6, D2, D6
4_a	34.3, CH_2	3.74, dd (2.5; 16.5)	4_b	3, 4a, 8a, A1	4_b, A2, A6
4_b	34.3, CH_2	3.04, dd (5.2; 16.7)	3, 4_a	3, A1, 4a	4_a
4a	144.0, C	-			
5	131.4, C	-			
6	137.5, CH	7.60, d (8.8)	7	4a, 8, 9	7, 10_a, 10_b
7	116.38, CH	6.74, d (8.8)	6	5, 8a	6
8	166.3, C	-			
8a	118.7, C	-			
9	203.8, C	-			
10_a	49.5, CH_2	3.51, dd (7.7; 16.2)	10_b, 11	9, 11, B1, C1	10_b, 11, B2, B6
10_b	49.6, CH_2	3.37, dd (7.9; 16.2)	10_a, 11	9, 11, B1, C1	10_a, 11, C2, C6
11	47.5, CH	4.40, t (7.6)	10_a, 10_b	9, 10, B1, B2, B6, C1, C2, C6	10_a, 10_b, B2, B6, C2, C6

12	142.0, CH	8.02, s		1, 2, 3, D2, D6	A6, D2, D6
A1	134.4, C	-			
A2	112.2, CH	6.76, d (1.7)		3, A4, A6	OCH_3 (A3), 4_a
A3	149.2, C	-			
A4	145.9, C	-			
A5	116.3, CH	6.62, d (8.1)	A6	A1, A3	A6
A6	121.1, CH	6.47, dd (1.7; 8.2)	A5	3, A2, A4	A5, 4_a
B1	137.7, C	-			
B2	112.8, CH	6.71, d (1.9)		11, B6	10_a, 11, OCH_3 (B3)
B3	148.8, C	-			
B4	145.9, C	-			
B5	116.1, CH	6.67, d (8.2)	B6	B1	
B6	121.3, CH	6.60, dd (1.9; 8.0)	B5	11, B2, B4	10_a, 11
C1	137.4, C	-			
C2	112.6, CH	6.69, d (1.9)		11, C4, C6	10_b, 11, OCH_3 (C3)
C3	148.8, C	-			
C4	146.4, C	-			
C5	116.04, CH	6.65, d (8.1)	C6	C3	C6
C6	121.1 CH	6.56, dd (2.0; 8.2)	C5	11, C2, C4	10_b, 11, C5
D1	127.9, C	-			
D2	114.2, CH	6.89, d (1.3)		12, D4, D6	12, OCH_3 (D3)
D3	148.8, C	-			
D4	150.1, C	-			
D5	116.6, CH	6.78, d (8.2)	D6	D1, D3	
D6	126.6, CH	6.95, d (8.2)	D5	12, D4, D2	12
OCH_3 (A3)	56.2, CH_3	3.63, s		A3	
OCH_3 (B3)	56.3, CH_3	3.71, s		B3	
OCH_3 (C3)	56.3, CH_3	3.70, s		C3	
OCH_3 (D3)	56.1, CH_3	3.56, s		D3	
